# Fbxo9 functions downstream of Sox10 to determine neuron-glial fate choice in the dorsal root ganglia through Neurog2 destabilization

**DOI:** 10.1073/pnas.1916164117

**Published:** 2020-02-06

**Authors:** Jessica Aijia Liu, Andrew Tai, Jialin Hong, May Pui Lai Cheung, Mai Har Sham, Kathryn S. E. Cheah, Chi Wai Cheung, Martin Cheung

**Affiliations:** ^a^Department of Anaesthesiology, Li Ka Shing Faculty of Medicine, The University of Hong Kong, Hong Kong, China;; ^b^School of Biomedical Sciences, Li Ka Shing Faculty of Medicine, The University of Hong Kong, Hong Kong, China

**Keywords:** Sox10, Neurog2, Fbxo9, neural crest progenitors

## Abstract

The transcription factor Sox10 is a key regulator in the fate determination of a subpopulation of multipotent trunk neural crest (NC) progenitors toward glial cells instead of sensory neurons in the dorsal root ganglia (DRG). However, the mechanism by which Sox10 regulates glial cell fate commitment during lineage segregation remains poorly understood. In our study, we showed that the neurogenic determinant Neurogenin 2 (Neurog2) exhibited transient overlapping expression with Sox10 in avian trunk NC progenitors, which progressively underwent lineage segregation during migration toward the forming DRG. Gain- and loss-of-function studies revealed that the temporary expression of Neurog2 was due to Sox10 regulation of its protein stability. Transcriptional profiling identified Sox10-regulated F-box only protein (Fbxo9), which is an SCF (Skp1-Cul-F-box)-type ubiquitin ligase for Neurog2. Consistently, overexpression of Fbxo9 in NC progenitors down-regulated Neurog2 protein expression through ubiquitination and promoted the glial lineage at the expense of neuronal differentiation, whereas Fbxo9 knockdown resulted in the opposite phenomenon. Mechanistically, we found that Fbxo9 interacted with Neurog2 to promote its destabilization through the F-box motif. Finally, epistasis analysis further demonstrated that Fbxo9 and probably other F-box members mediated the role of Sox10 in destabilizing Neurog2 protein and directing the lineage of NC progenitors toward glial cells rather than sensory neurons. Altogether, these findings unravel a Sox10–Fbxo9 regulatory axis in promoting the glial fate of NC progenitors through Neurog2 destabilization.

## Significance

The neuron–glial lineage segregation of NC progenitors forming the DRG is essential for peripheral nervous system development. However, hierarchical transcriptional control does not explain how this lineage bifurcation occurs. Here, we provide in vivo evidence that a glial lineage specifier, Sox10, inhibits neurogenesis via a degradation mechanism. A ubiquitin-ligase, Fbxo9, acting downstream of Sox10 destabilizes the proneural Neurog2 protein, leading to the loss of neurogenic capacity in NC progenitors while leaving their glial potential intact. Functional inhibition of Fbxo9 and possibly other members of the F-box family lead to prolonged expression of Neurog2 protein that causes both the expansion of NC progenitors and precocious neuronal differentiation. Our results unravel a previously unrecognized mechanism in neuron–glial cell fate decision.

A long-standing question in the field of developmental biology is how a pool of multipotent progenitors can be established and maintained and become fate-restricted to ultimately dictate the identity of the differentiated functional cell types during the development of tissues in multicellular organisms. Despite considerable progress in ascribing key genes to specific lineages, the molecular mechanisms employed by individual progenitor cells during lineage commitment remain largely unknown. For example, the neural crest (NC), a migratory population arising from the neural plate border during early embryogenesis, has the ability to differentiate into sensory neurons and satellite glial cells in the dorsal root ganglia (DRG), which can serve as an excellent model for studying fate determination in the nervous system (1).

During vertebrate embryogenesis, Sox10, a member of the SOXE transcription factor family (Sox8, Sox9, Sox10), is initially expressed in premigratory and early migratory NC cells (NCCs) and later becomes restricted in satellite glial cells and Schwann cell precursors, whereas it is down-regulated in the sensory DRG and in autonomic and enteric neurons (2, 3). Consistent with its early expression in multipotent NC stem cells or progenitor populations, constitutive expression of Sox10 in a clonal culture of mouse NC stem cells maintained their multipotency by preserving both neurogenic and gliogenic capacity upon appropriate lineage-commitment signals (4). Likewise, overexpression of Sox10 maintained most of the avian NCCs in an undifferentiated state and inhibited them from colonizing sites of neurogenesis. Nevertheless, a small proportion of Sox10-transfected cells were still compatible with glial differentiation (5). These findings were further supported by several reports that Sox10 deletion or inactivating mutations in mice had no effect on the initial formation and emigration of NCCs, probably due to the compensatory functions of Sox8 and/or Sox9, but later affected the maintenance of multipotency, leading to the loss of both glial and neuronal lineages (6–9). Homozygous *Sox10* mouse mutants lacking satellite glial formation exhibited subsequent degeneration of DRG neurons due to loss of glial trophic support (9, 10). However, previous studies in zebrafish embryos demonstrated that Sox10 was transiently expressed in the sensory neuron lineage and was required for specifying sensory neuron precursors by activating the expression of proneural basic helix–loop–helix (bHLH) transcription factor gene *Neurogenin 1* (*Neurog1*) independently of glial support (11). In agreement with this, Neurog1 was sufficient to promote DRG neuron formation in wild-type embryos and rescued the loss of sensory neurons in Sox10 morphants (12, 13). Together, these data suggest that the mechanisms employed by Sox10 in the fate determination of NC progenitors to form sensory neurons versus satellite glial cells could be species-specific.

Similarly, forced expression of either Neurog2 or Neurog1 biased chick premigratory NCCs to localize to the dorsal root sensory ganglia, and also converted nonsensory NC derivatives into the sensory neuron fate (14). In mouse, Neurog2 and Neurog1 are required in the early and later phases for the formation of different sensory neuron subtypes, respectively (15). These findings are in agreement with lineage-tracing experiments in mice that found that Neurog2 was transiently expressed in a subset of Sox10^+^ NC migratory streams and in the majority of its descendants that exhibited strong lineage bias toward sensory neurons versus satellite glial cells (16). Moreover, several reports have demonstrated that Neurog2 is a highly unstable protein degraded by the ubiquitin–proteasome system (UPS) (17–19), rendering its expression transient, yet it is still capable of activating downstream events to specify sensory neurogenesis. However, the factors that mediate the degradation process remain to be identified. Based on these findings, we speculate that the initial coexpression of Neurog2 and Sox10 could mark bipotent neuron/glial precursors, which gradually segregate to form Sox10^+^ glial and Neurog2-derived sensory lineages in the DRG, but the molecular mechanisms of how this lineage segregation occurs remain poorly understood.

The F-box only protein 9 (Fbxo9) is a member of the F-box protein family and functions as a substrate-recognition subunit of the Skp1–Cullin1–F-box protein E3 ligase complex, and plays pivotal roles in ubiquitination and in the subsequent degradation of target proteins (20). It has been shown that Fbxo9 is augmented in human coronary arterial smooth muscle cells under high glucose culture and in the vessels of streptozotocin-induced diabetic rat, leading to UPS-mediated BK-β_1_ degradation. In addition, Fbxo9 was shown to be required for adipocyte differentiation through modulating the level of peroxisome proliferator-activated receptor gamma protein (21, 22). Another study showed that, in response to growth factor deprivation, Fbxo9-mediated ubiquitination of telomere maintenance 2 (Tel2) and Tel2 interacting protein 1 (Tti1) inactivated mTORC1, but activated the PI(3)K/TORC2/Akt pathway to promote survival in multiple myeloma (23). To date, we know very little about the regulation and function of Fbxo9 during development.

Here, we investigated the molecular mechanisms of how Sox10^+^ NC progenitors with transient expression of neurogenic determinant Neurog2 can acquire glial cell fate over sensory neuron fate in avian DRG. Overexpression of Sox10 promoted ubiquitin-mediated degradation of Neurog2 concomitant with reduced neurogenic potency of NCCs, whereas Neurog2 protein persisted in the absence of Sox10 function. Gene expression profiling using RNA sequencing identified Fbxo9 as a downstream transcriptional target of Sox10 involved in the ubiquitination of Neurog2 through its F-box motif. Consistently, Fbxo9 was both required and sufficient for the differentiation of bipotent NC progenitors into satellite glial cells instead of sensory neurons. Epistasis analysis further showed that Fbxo9 knockdown or functional inhibition of other Fbxo members in Sox10-overexpressing cells restored the formation of sensory neurons in the DRG. Altogether, our studies revealed a mechanism by which Sox10 activates Fbxo9 expression to destabilize Neurog2 protein by ubiquitination, leading to segregation of NC progenitors into satellite glial cells but not sensory neurons in the DRG.

## Results

### Segregation of Sox10- and Neurog2-Expressing Migratory NCCs.

**Fig. 1.**
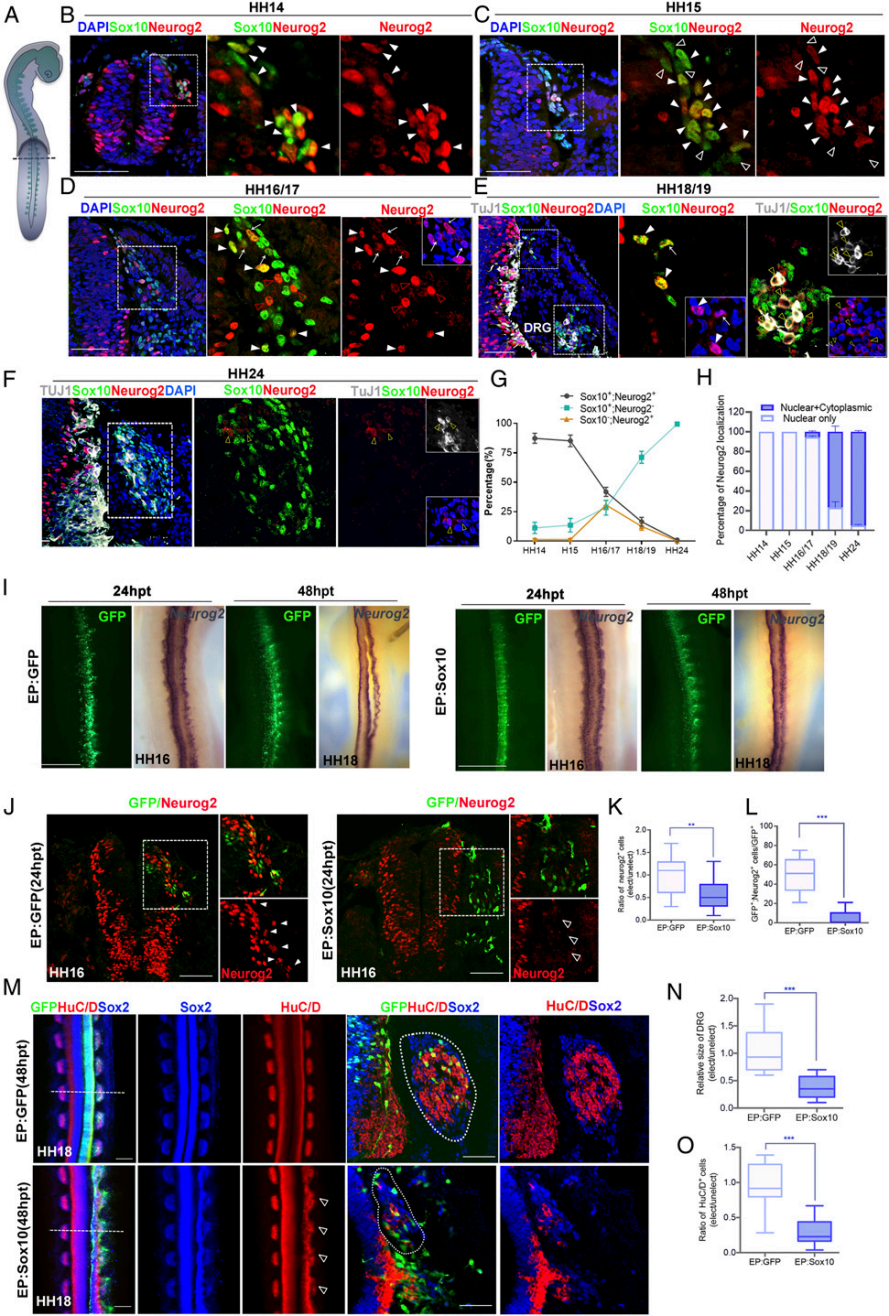
Overexpression of Sox10 negatively regulates Neurog2 protein expression. Immunofluorescence for Sox10 and Neurog2 in the transverse sections of the trunk neural tube (*A*) from chicken embryos at (*B*) HH14, (*C*) HH15, (*D*) HH16/17, (*E*) HH18/19, and (*F*) HH24. The magnified areas are marked with dashed boxes. (*B*–*E*) Solid white arrowheads indicate Sox10^+^Neurog2^+^ (nuclear) cells. (*C*) Open arrowheads indicate cells expressing Sox10 alone. (*D* and *E*) White arrows indicate Sox10^+^Neurog2^+^ (nuclear+cytoplasmic). (*D*) Red open arrowheads indicate cells expressing Neurog2 (nuclear) alone. (*E* and *F*) TuJ1 was detected in the dorsal root ganglia (DRG) on HH18/19 and HH24. Yellow open arrowheads indicate cells expressing both Neurog2 (nuclear+cytoplasmic) and TuJ1 together. (*G*) Graph showing the percentage of cells expressing Sox10 or Neurog2 alone and both proteins in the total number of cells positive for the indicated markers at the indicated stages. Average number of cells counted from at least 15 sections from 3 embryos per stage. (*H*) Graph showing the percentage of cells with nuclear Neurog2 or nuclear+cytoplasmic Neurog2 in the total number of Neurog2^+^ cells. (*I*) In situ hybridization for *Neurog2* on embryos electroporated (EP) with GFP vector control (*n* = 5) or Sox10 (*n* = 5) at 24 and 48 hpt. (*J*) Immunofluorescence for Neurog2 on transverse sections of embryos electroporated with the indicated constructs. The magnified areas are marked with dashed boxes. Solid white arrowheads indicate endogenous Neurog2 expression, and open arrowheads indicate loss of Neurog2 expression. (*K*) Graph showing ratio of Neurog2^+^ cells between the electroporated and unelectroporated sides of embryos treated with the indicated constructs. (*L*) Quantification of the number of Neurog2^+^ cells in embryos transfected with the indicated constructs. (*M*) Immunofluorescence for HuC/D and Sox2 in embryos electroporated with GFP (*n* = 6) or Sox10 (*n* = 7). Dotted lines indicate plane of sectioning. Open arrowheads indicate DRG dysplasia. The border of the DRG is marked by dotted lines. (*N*) Graph showing fold differences in the size of the DRG between the electroporated and unelectroporated sides of embryos treated with the indicated constructs. (*O*) Graph showing ratio of HuC/D^+^ cells between the electroporated and unelectroporated sides of embryos treated with the indicated constructs. Error bars ± SEM (**P < 0.01, ***P < 0.001). (Scale bars: embryos, 20 μm; sections, 50 μm.)

To investigate how Sox10 and Neurog2 contribute to lineage segregation of NC progenitors, we first examined their expression patterns in avian migrating trunk NCCs (Fig. 1*A*) by immunofluorescence with antibodies against Sox10 and Neurog2. The majority of nuclear Neurog2 was initially detected in a subset of Sox10^+^ early-migrating NCCs at Hamburger and Hamilton (HH) stages 14/15 (Fig. 1 *B*, *C*, *G*, and *H*), at the beginning of the first wave of neurogenesis (14, 24). By HH16/17, the number of Sox10^+^/Neurog2^+^ cells gradually decreased, along with increasing numbers of migrating Sox10^+^ or Neurog2^+^ NCCs (Fig. 1 *D* and *G*). In addition, we observed a few Sox10^+^ cells expressing Neurog2 with a dual nuclear and cytoplasmic localization (Fig. 1 *D* and *H*). At HH18/19, when most NCCs had colonized at the prospective DRG (24), only a small portion of Sox10^+^/Neurog2^+^ (nuclear) cells appeared in the dorsal pole, and the majority of cells expressing Sox10 alone were detected in the developing DRG, where expression of pan-neuronal marker TuJ1 became prominent in a subset of neuronal precursors with nuclear and cytoplasmic localization of Neurog2 (Fig. 1 *E*, *G*, and *H* and *SI Appendix*, Fig. S1). This observation is consistent with previous studies that found that the first differentiated neurons in the core of the ganglion were derived from the earliest wave of Neurog2^+^ migrating NCCs (24) and that the nuclear-to-cytoplasmic shuttling of Neurog2 indicates that it may no longer be required for further differentiation processes. By HH24, Sox10^+^ cells had migrated to the forming DRG, where Neurog2 expression was down-regulated with residual protein in both the nucleus and cytoplasm of TuJ1^+^ cells (Fig. 1 *F*–*H*). These expression studies revealed an initial coexpression of Sox10 and nuclear Neurog2 in bipotent neuroglial NC progenitors followed by relocalization of Neurog2 from the nucleus to cytoplasm at the point of lineage segregation.

### Overexpression of Sox10 Down-Regulates Neurog2 Protein Expression and Reduces Neurogenic Potency of NC Progenitors.

As Sox10 is required for specifying peripheral glia (9), we next examined the possibility that Sox10 determines glial lineage by repressing the expression of Neurog2 at the transcriptional or translational levels. To overexpress Sox10, full-length *Sox10* cDNA in a pCIG-IRES-nls-EGFP (pCIG) vector was electroporated into the trunk of hemineural tube at HH11/12 prior to the initiation of Neurog2 expression. Embryos were analyzed for Neurog2 expression 24 and 48 h posttransfection (hpt). Similar to the vector control, overexpression of Sox10 did not affect *Neurog2* transcript levels at 24 and 48 hpt, by which time NCCs had completed migration and condensed to form the DRG (Fig. 1*I*). In contrast, ectopic expression of Sox10 down-regulated Neurog2 protein expression in migrating NCCs at 24 and 48 hpt compared to the unelectroporated side and vector control, where cells coexpressing GFP and Neurog2 were detected (Fig. 1 *J*–*L* and *SI Appendix*, Fig. S2 *A*–*C*). These results suggest that overexpression of Sox10 negatively regulates Neurog2 protein expression.

We then examined the effects of Sox10 overexpression on lineage differentiation by staining the transfected embryos for Sox2, which marked both proliferating neuron–glial progenitors along the DRG perimeter and glial precursors in the core (25), as well as with a definitive neuronal marker, HuC/D. Consistent with the ongoing neuronal differentiation, we observed that the majority of the NC-derived cells transfected with control vector expressed HuC/D but not Sox2 within the DRG of electroporated embryos (Fig. 1 *M* and *O*). In contrast, most of the Sox10-overexpressing cells migrated to the periphery lateral to the neural tube without colonizing the DRG. There was only a small portion of transfected cells positive for HuC/D in the core, resulting in an overall smaller sized DRG compared to the vector control (Fig. 1 *M*–*O*). Altogether, these data suggested that Sox10 overexpression down-regulated Neurog2 protein, partly contributing to the reduced potency of migratory NCCs for differentiation into sensory neurons in the DRG.

### Sox10 Destabilizes Neurog2 through Ubiquitination.

**Fig. 2.**
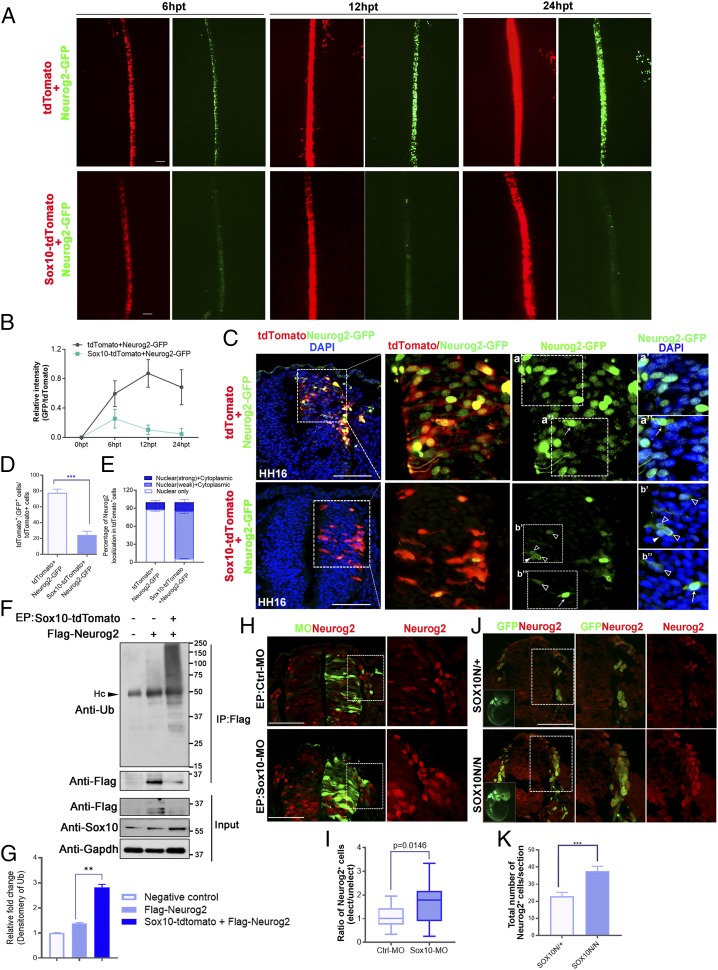
Sox10 regulates Neurog2 protein stability. (*A*) Dorsal view of chicken embryos transfected with tdTomato+Neurog2-GFP (*n* = 6) or Sox10-tdTomato+Neurog2-GFP (*n* = 7) at 6, 12, and 24 hpt. (*B*) Quantification of relative fluorescence intensities (GFP/tdTomato) in embryos transfected with the indicated constructs at each time point. (*C*) Cross-sections of embryos electroporated with the indicated constructs. Nuclei were counterstained with DAPI. (*a*′, *a*″, *b*′, and *b*″). The magnified areas are marked with dashed boxes. (*a*′) The majority of Neurog2-GFP proteins were localized in the nucleus of cells expressing vector control. (*a*″ and *b*″) White arrows indicate cells expressing strong nuclear+cytoplasmic Neurog2-GFP. (*b*′) White solid arrowhead indicates nuclear localization of Neurog2-GFP only. (*b*′ and *b*″) Open arrowheads indicate cells expressing weak nuclear+cytoplasmic Neurog2-GFP. (*D*) Quantification of the number of tdTomato^+^GFP^+^ cells in each treatment. (*E*) Graph showing the percentage of cells with nuclear Neurog2-GFP only, nuclear (strong)+cytoplasmic Neurog2-GFP, and nuclear (weak)+cytoplasmic Neurog2-GFP in the total number of GFP^+^ cells in embryos treated with the indicated constructs at 24 hpt. (*F*) Well-transfected chicken embryos with the indicated constructs (*n* = 10 per treatment) were subjected to immunoprecipitation (IP) with anti-Flag and blotted with anti-Ub and anti-Flag. A total of 20% of the total input was blotted with anti-Sox10 and anti-Flag. Gapdh served as a loading control. (*G*) Densitometric quantification of the levels of Flag-Neurog2 conjugated to ubiquitin (Ub) in each treatment relative to the control. (*H*) Cross-sections of embryos electroporated with control morpholino (Ctrl-MO; *n* = 8) or Sox10-MO (*n* = 8) at 24 hpt. The magnified areas are marked with dashed boxes. (*I*) Graph showing ratio of Neurog2^+^ cells between electroporated and unelectroporated sides of embryos transfected with the indicated constructs. (*J*) Immunofluorescence for GFP and Neurog2 on transverse sections of Sox10N/+ and Sox10N/N mouse embryos at E9.5. (*K*) Quantification of the number of Neurog2^+^ migratory NCCs of Sox10N/+ (*n* = 5) and Sox10N/N mutants (*n* = 5) at E9.5. Error bars ± SEM (**P < 0.01, ***P < 0.001). Hc, heavy chains. (Scale bars: embryos, 20 μm; sections, 50 μm.)

We further elucidated the inhibitory effects of Sox10 overexpression on Neurog2 protein level by using exogenous Neurog2. Misexpressed Sox10 in pCIG-IRES-tdTomato vector with Neurog2 tethered by C-terminal fusion of GFP (Neurog2-GFP) was electroporated into the hemineural tube of HH11 chicken embryos. The relative Neurog2-GFP fluorescence intensity was measured in cells expressing tdTomato vector control or Sox10-tdTomato at 6, 12, and 24 hpt. Embryos transfected with the tdTomato vector control showed a gradual increase in Neurog2-GFP fluorescence intensity from 6 to 24 hpt, whereas embryos treated with Sox10 showed dramatically diminished Neurog2-GFP fluorescence intensity from 6 to 24 hpt (Fig. 2 *A* and *B*). Consistently, detailed examination of a cross-section of transfected embryos at 24 hpt revealed overlapping expression of tdTomato vector control with the expression of Neurog2-GFP that was predominantly nuclear (Fig. 2 *C*–*E*). In addition to reduced number of Neurog2-GFP^+^ cells, most of the Sox10-overexpressing cells exhibited nuclear (weak) and cytoplasmic localization of Neurog2-GFP, whereas a small portion of them harbored nuclear (strong) and cytoplasmic Neurog2-GFP (Fig. 2 *C*–*E*). These findings were consistent with our previous observation of endogenous Neurog2 protein with dual subcellular localization in Sox10-expressing cells (Fig. 1 *D*, *E*, and *H*). The relocalization of ectopic Neurog2 from the nucleus to the cytoplasm suggests the possibility that overexpression of Sox10 promotes ubiquitination of Neurog2, which could occur in both compartments as evidenced by another short-lived bHLH transcription factor, MyoD (26). In agreement with this, at 9 hpt, overexpression of Sox10 enhanced the level of ubiquitin (Ub)-conjugated ectopic Flag-Neurog2, which exhibited a ladder of high-molecular-weight bands that may correspond to polyubiquitinated forms of the protein compared to low levels of ubiquitination in embryos electroporated with Flag-Neurog2 alone. In contrast, ubiquitinated protein bands were barely detectable in Flag pull-down lysates from wild-type embryos (Fig. 2 *F* and *G*). These results confirmed that the strong high-molecular-weight smear of bands represented ubiquitinated Flag-Neurog2. Consistently, immunoblotting with anti-Flag showed that the level of Flag-Neurog2 expression (∼35 kDa) was higher in embryos treated with Flag-Neurog2 alone than in embryos treated with Sox10-tdTomato+Flag-Neurog2 (Fig. 2*F*). On the contrary, increased numbers of Neurog2^+^ cells were detected in chicken embryos treated with Sox10 morpholino (MO) (27) and in Sox10-NGFP–knockout mice (Sox10N/N) with the N-terminal domain of Sox10 fused to an EGFP reporter (28) when compared to MO control (Ctrl-MO) and Sox10 heterozygous (Sox10N/+) groups, respectively (Fig. 2 *H*–*K*). Taken together, these findings suggest that Sox10 regulates Neurog2 protein stability.

### Sox10 Regulates Fbxo9 Expression.

**Fig. 3.**
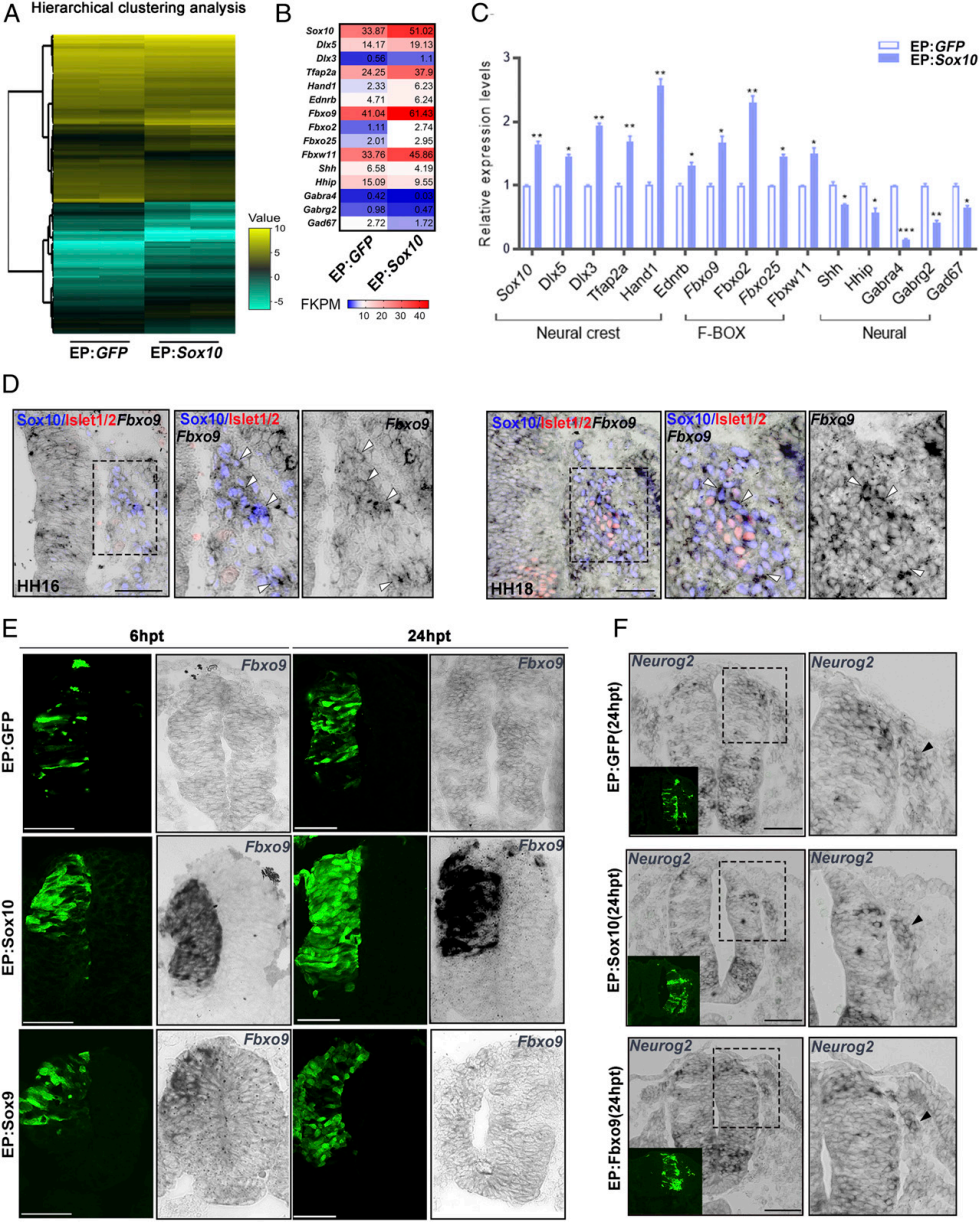
Sox10 regulates Fbxo9 expression. (*A*) Hierarchical clustering of biological replicates of differentially expressed genes in GFP^+^ cells sorted from embryos treated with vector control and Sox10 overexpression. (*B*) Heat map of gene expression differences between vector control and Sox10 overexpression. (*C*) qPCR validation of differentially expressed genes between vector control and Sox10 overexpression. (*D*) In situ hybridization for *Fbxo9* on cross-sections of embryos at HH16 and HH18 followed by immunofluorescence for Sox10 and Islet1/2. The magnified regions are marked with dashed boxes. White solid arrowheads indicate cells coexpressing *Fbxo9* and Sox10. (*E*) In situ hybridization for *Fbxo9* on cross-sections of embryos electroporated with vector control (*n* = 5), Sox10 (*n* = 6), or Sox9 (*n* = 7) at 6 and 24 hpt. (*F*) In situ hybridization for *Neurog2* on cross-sections of embryos treated with vector control (*n* = 5), Sox10 (*n* = 6), and Sox9 (*n* = 7) at 24 hpt. Black arrowheads indicate endogenous *Neurog2* mRNA expression. Error bars ± SEM (*P < 0.05, **P < 0.01, ***P < 0.001). (Scale bars, 50 μm.)

To identify candidate factors that mediate Sox10 regulation of Neurog2 stability, we performed RNA sequencing (RNA-seq) on Sox10-overexpressing cells sorted from electroporated chicken embryos at 9 hpt. Hierarchical clustering showed good correlation of expression levels between the two biological replicates, which confirmed the robust cell isolation method (Fig. 3*A*). The mean of the replicates showed significant differences in the expression levels of subsets of genes between Sox10 overexpression and control (Fig. 3*B* and Datasets S1 and S2) (29). Among them, *Dlx5*, *Dlx3*, *Tfap2a*, *Hand1*, *and Ednrb* implicated in NC development were up-regulated, whereas neural patterning molecule *Sonic hedgehog* (*Shh*), its negative regulator *Hhip*, and several neuronal markers (*Gabra4*, *Gabrg2*, *and Gad67*) were down-regulated (Fig. 3 *B* and *C*). This was in agreement with studies that found that ectopic expression of Sox10 induced NCC fate at the expense of neurogenesis (5, 7, 30). In addition, Sox10 also induced up-regulation of the expression of several F-box family members, including *Fbxo2* and *Fbxo25*, but their expression in the DRG was barely detectable (Fig. 3*C* and *SI Appendix*, Fig. S3). We found that *Fbxw11* was expressed in DRG neurons, but did not appear to coincide with the role of Sox10 in specifying glial lineage (*SI Appendix*, Fig. S3). We selected the *Fbxo9* gene for further studies because it showed overlapping expression with Sox10 in migratory NCCs at HH16, but not with Islet1/2^+^ sensory neurons in the developing DRG at HH18 (Fig. 3*D*). Consistent with the RNA-seq data, ectopic *Fbxo9* mRNA was detected in most of the Sox10-overexpressing cells at 6 and 24 hpt compared to the vector control, which did not induce *Fbxo9* expression (Fig. 3*E*), indicating that the effect was cell-autonomous and specific. In contrast, the ability of Sox9 overexpression to induce ectopic *Fbxo9* expression was less pronounced at 6 hpt, and no induction was detected at 24 hpt (Fig. 3*E*). This could be due to an insufficient level of *Sox10* mRNA induced by Sox9 overexpression at 6 and 24 hpt to trigger ectopic *Fbxo9* expression (*SI Appendix*, Fig. S4 *A* and *B*). Nevertheless, these results confirmed the RNA-seq data that Sox10 is sufficient to induce *Fbxo9* expression.

### Fbxo9 Regulates Neurog2 Protein Levels and Glial Cell Fate Determination.

**Fig. 4.**
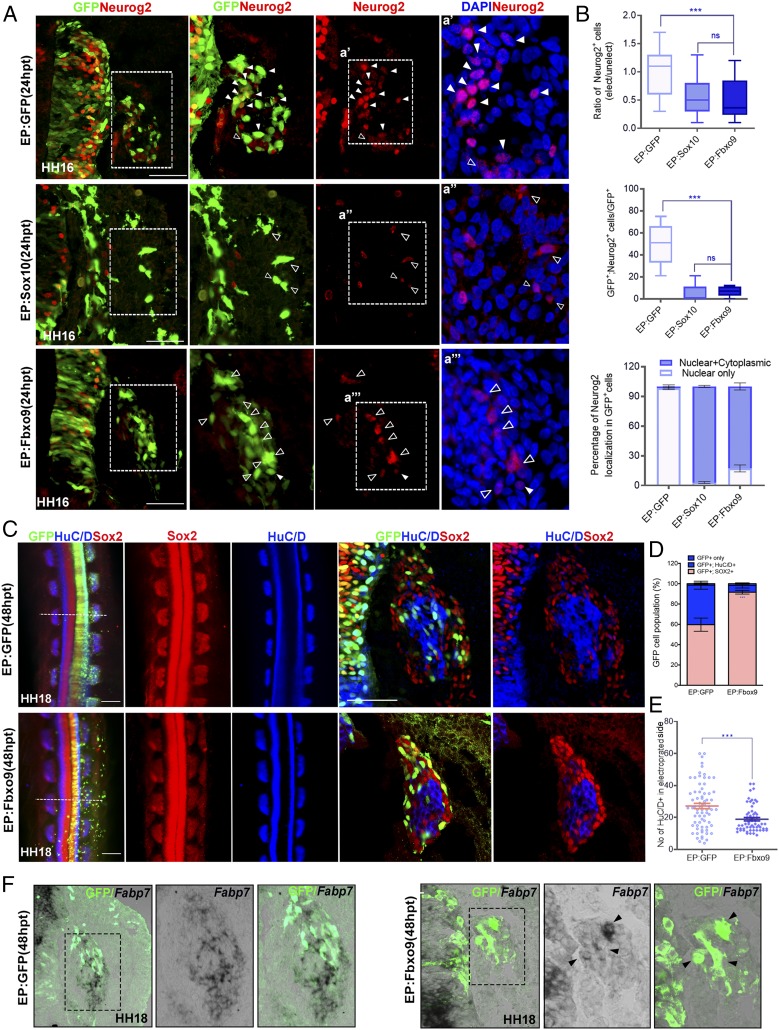
Overexpression of Fbxo9 reduces Neurog2 protein expression and directs NCCs to differentiate into glial lineage. (*A*) Immunofluorescence for Neurog2 on transverse sections of embryos treated with GFP (*n* = 6), Sox10 (*n* = 6), and Fbxo9 (*n* = 7) at 24 hpt (or HH16). (*a*′, *a*″, and *a*″′) The magnified areas are marked with dashed boxes. Nuclei were counterstained with DAPI. Solid white and open arrowheads indicate cells with nuclear Neurog2 and nuclear+cytoplasmic Neurog2 expression, respectively. (*B*) Graph showing ratio of Neurog2^+^ cells between electroporated and unelectroporated sides of neural tubes treated with the indicated constructs. Quantification of the number of GFP^+^Neurog2^+^ cells in embryos treated with the indicated constructs. Graph showing the percentage of cells with nuclear Neurog2 only and nuclear+cytoplasmic Neurog2 in the total number of GFP^+^ cells from embryos treated with the indicated constructs. (*C*) Whole-mount immunofluorescence for GFP, HuC/D, and Sox2 on embryos treated with GFP (*n* = 6) or Fbxo9 (*n* = 7) at 48 hpt. White dotted lines indicate the plane of sectioning. (*D*) Quantification of the number of cells expressing either GFP^+^ alone, GFP^+^HuC/D^+^, or GFP^+^Sox2^+^ in embryos treated with the indicated constructs. (*E*) Quantification of the number of HuC/D^+^ cells in the electroporated side of embryos treated with the indicated constructs. (*F*) In situ hybridization for *Fabp7* expression on cross-sections of embryos treated with the indicated constructs at 48 hpt followed by immunofluorescence for GFP. The magnified areas are marked with dashes boxes. Black arrowheads indicate cells coexpressing GFP and *Fabp7*. Error bars ± SEM. ns, nonsignificant (***P < 0.001). (Scale bars: embryos, 10 μm; sections, 50 μm.)

**Fig. 5.**
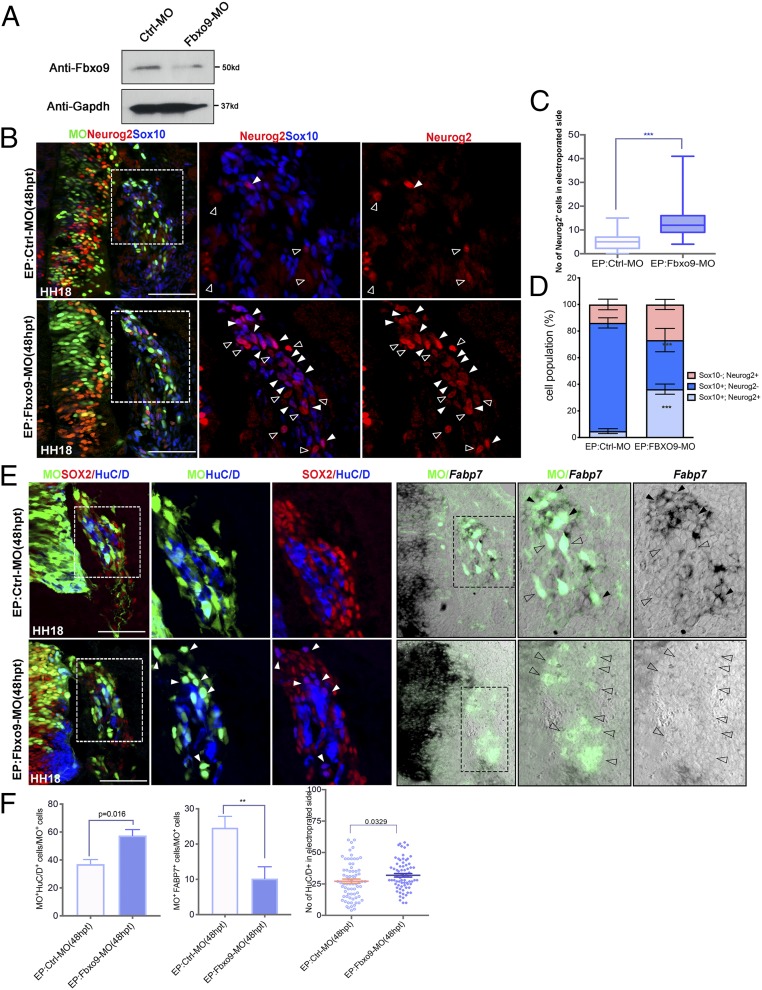
Fbxo9 knockdown leads to persistent Neurog2 protein expression and inhibits glial differentiation. (*A*) Lysates from chicken embryos treated with Ctrl-MO (*n* = 10) and Fbxo9 MO (*n* = 10) at 24 hpt were subjected to Western blotting for Fbxo9. GAPDH served as a loading control. (*B*) Immunofluorescence for Neurog2 and Sox10 on cross-sections of embryos treated with Ctrl-MO (*n* = 8) and Fbxo9 MO (*n* = 8) at 48 hpt. Solid white and open arrowheads indicate cells expressing both Sox10/Neurog2 and Neurog2 alone, respectively. The magnified areas are marked with dashed boxes. (*C*) Quantification of Neurog2^+^ cells in the electroporated side of embryos treated with the indicated constructs. (*D*) Quantification of the number of cells expressing Neurog2 alone, Sox10 alone, and both together in embryos treated with the indicted constructs. (*E*) Immunofluorescence for Sox2 and HuC/D on cross-sections of embryos electroporated with the indicated constructs at 48 hpt. White arrowheads indicate cells expressing both Sox2 and HuC/D. Adjacent sections were subjected to in situ hybridization for *Fabp7* followed by V5 immunofluorescence to mark the MO transfected cells. Black arrowheads indicate cells coexpressing MO and *Fabp7*, and open arrowheads indicate cells expressing MO alone in the DRG. (*F*) Quantification of the number of MO^+^HuC/D^+^ or MO^+^*Fabp7*^+^ cells in embryos treated with the indicated constructs. Quantification of the number of HuC/D^+^ cells in the electroporated side of embryos treated with the indicated constructs. Error bars ± SEM (**P < 0.01, ***P < 0.001). (Scale bars, 50 μm.)

We next examined whether Fbxo9 overexpression affected Neurog2 protein expression in a similar manner to Sox10. As observed in embryos treated with Sox10, overexpression of pCIG-Fbxo9 full-length cDNA at 24 hpt did not alter *Neurog2* mRNA, but reduced its protein expression in migratory NCCs (Figs. 3*F* and 4 *A* and *B*). The residual amount of Neurog2 protein was detected in the nucleus and the cytoplasm of both Fbxo9- and Sox10-expressing cells compared to predominant nuclear localization of Neurog2 in the vector control (Fig. 4 *A* and *B*). In contrast, the number of HuC/D^+^ cells within the DRG core was reduced to lesser degree in embryos treated with Fbxo9 than in embryos electroporated with Sox10 (Fig. 4 *C*–*E* compared to Fig. 1 *M* and *O*). We found that Fbxo9-expressing cells predominantly localized in the periphery of the DRG expressed Sox2 and that some expressed satellite glial marker *Fatty acid binding protein 7* (*Fabp7*), indicating that these cells either remained undifferentiated or were differentiated into satellite glial cells (Fig. 4 *C*, *D*, and *F*). To investigate whether Fbxo9 was required in the regulation of Neurog2 protein stability and in fate determination in the DRG, we performed Fbxo9 knockdown by electroporating a translational-blocking morpholino (Fbxo9-MO) into the trunk neural tube of HH11 embryos, while control MO (Ctrl-MO) served as a negative control (Fig. 5*A*). Analysis of embryos treated with Fbxo9-MO at 48 hpt revealed increased numbers of cells coexpressing Neurog2 and Sox10 in the DRG, whereas Neurog2 expression was significantly diminished in the Ctrl-MO group (Fig. 5 *B*–*D*). Consistently, Fbxo9-MO resulted in more cell differentiation into HuC/D^+^ neurons instead of *Fabp7*^+^ glial cells when compared to the Ctrl-MO group (Fig. 5 *E* and *F*). Although Sox2 and HuC/D exhibited mutually exclusive expression in the DRG of embryos treated with Ctrl-MO, we observed that a few Sox2^+^ cells expressed HuC/D in the DRG of embryos treated with Fbxo9-MO (Fig. 5*E*), suggesting early onset of neuronal differentiation. These results indicate that Fbxo9 plays a role in regulating Neurog2 protein expression and glial differentiation.

### Fbxo9 Functions Downstream of Sox10 to Regulate Neurog2 Stability.

**Fig. 6.**
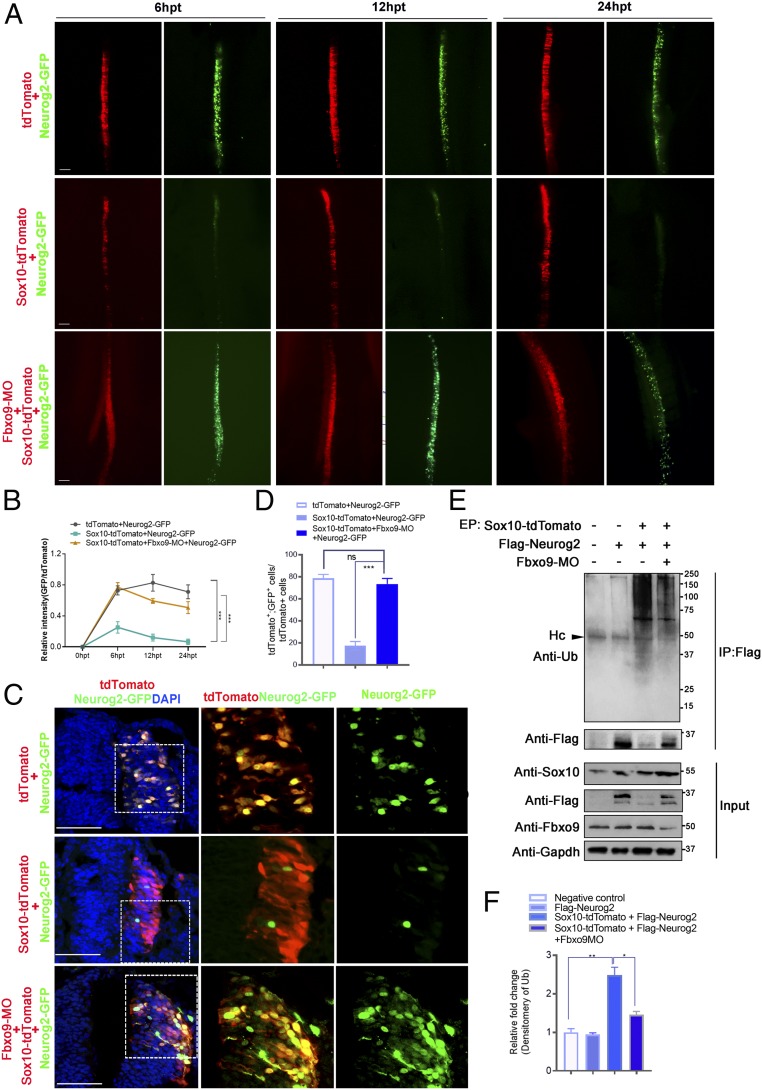
Fbxo9 mediates the role of Sox10 in Neurog2 protein stability. (*A*) Dorsal view of chicken embryos transfected with tdTomato+Neurog2-GFP (*n* = 7), Sox10-tdTomato+Neurog2-GFP (*n* = 8), and Sox10-tdTomato+Fbxo9-MO+Neurog2-GFP (*n* = 8) at 6, 12, and 24 hpt. (*B*) Quantification of fluorescence intensities (GFP/tdTomato) in embryos transfected with the indicated constructs. (*C*) Cross-sections of embryos electroporated with the indicated constructs at 24 hpt. The magnified areas are marked with dashed boxes. (*D*) Quantification of the number of tdTomato^+^GFP^+^ cells in each treatment group. (*E*) Well-transfected chicken embryos with the indicated constructs (*n* = 10 per treatment) were subjected to immunoprecipitation (IP) with anti-Flag2 and blotted with anti-Ub or anti-Flag. A total of 20% of the total input was blotted with anti-Sox10, anti-Flag, and anti-Fbxo9. Gapdh served as a loading control. (*F*) Densitometric quantification of the levels of Flag-Neurog2 conjugated to Ub in each treatment relative to the control. Hc, heavy chains. Error bars ± SEM. ns, nonsignificant (*P < 0.05, **P < 0.01, ***P < 0.001). (Scale bars: embryos, 20 μm; sections, 50 μm.)

We next examined whether Fbxo9 mediated some, if not all, of Sox10 functions involved in Neurog2 protein stability and glial cell formation in the DRG. The epistasis analysis revealed that cells expressing Sox10+Fbxo9-MO+Neurog2-GFP maintained robust GFP expression from 6 to 12 hpt with weaker GFP intensity at 24 hpt, whereas embryos treated with Sox10+Neurog2-GFP had significantly diminished GFP intensity from 6 to 12 hpt that was barely detectable at 24 hpt. As expected, persistent Neurog2-GFP expression was observed throughout the analysis in the vector control group (Fig. 6 *A*–*D*). Consistently, the level of Ub-conjugated Neurog2 was relatively lower in embryos electroporated with Sox10+Flag-Neurog2+Fbxo9-MO than in embryos treated with Sox10+Flag-Neurog2, which induced robust Neurog2 ubiquitination. In contrast, ubiquitinated Neurog2 was barely detectable in embryos electroporated with Flag-Neurog2 alone (Fig. 6 *E* and *F*). These results suggest that Fbxo9 partly mediates the role of SOX10 in destabilizing Neurog2 proteins.

### Overexpression of Fbxo9 Mutant Stabilizes Neurog2 Protein and Promotes Neuronal Differentiation in the DRG.

**Fig. 7.**
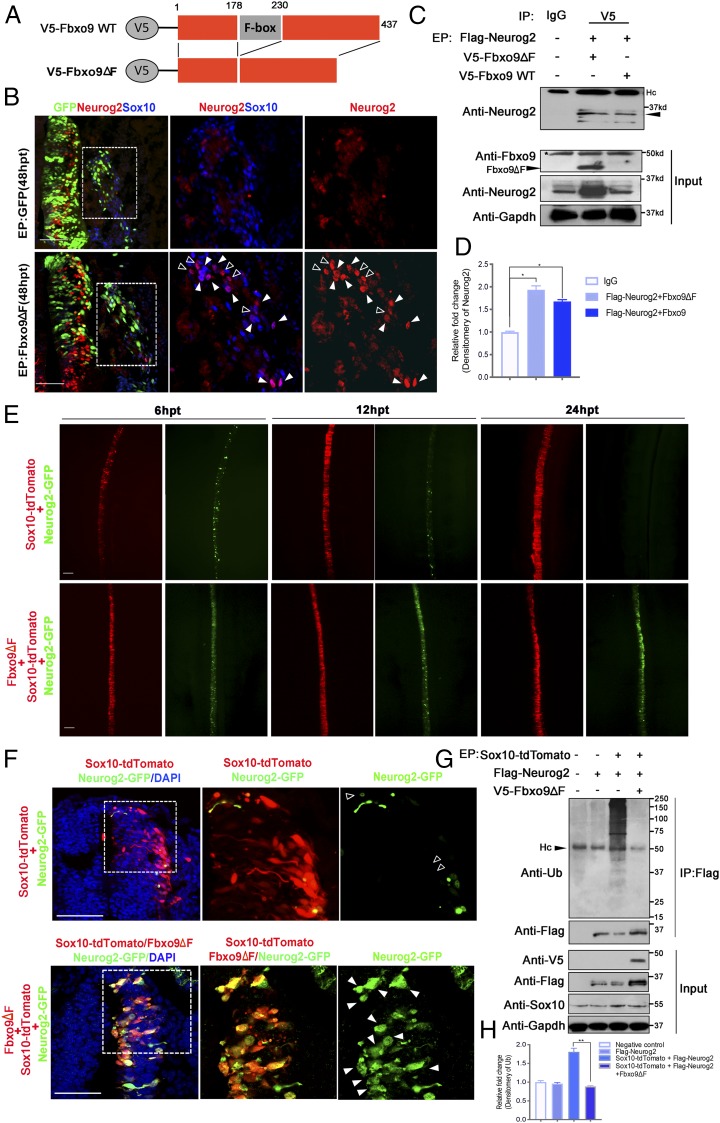
Mutant form of Fbxo9 stabilizes Neurog2 protein. (*A*) Structure of wild-type (WT) Fbxo9 and its mutant form without F-box domain. (*B*) Immunofluorescence for Neurog2 and Sox10 on cross-sections of embryos electroporated with GFP control (*n* = 7) or Fbxo9ΔF (*n* = 8) at 48 hpt. The magnified areas are marked with dashed boxes. Closed and open arrowheads indicate Sox10^+^Neurog2^+^ cells and cells expressing Neurog2 alone, respectively. (*C*) Well-transfected chicken embryos with the indicated constructs (*n* = 10 per treatment) were subjected to IP with anti-V5 and blotted with anti-Neurog2. A total of 20% of the total input was blotted with anti-Fbxo9 and anti-Neurog2 to detect both endogenous and ectopic levels of Fbxo9 and Neurog2 expression, respectively. Gapdh served as a loading control. Black arrowhead indicates endogenous and ectopic Neurog2 proteins pulled down by Fbxo9. Asterisk indicates endogenous Fbxo9 expression. (*D*) Densitometric quantification of the levels of immunoprecipitated endogenous and ectopic Neurog2 in each treatment relative to the control. (*E*) Dorsal view of chicken embryos transfected with Sox10-tdTomato+Neurog2-GFP (*n* = 7) and Sox10-tdTomato+Fbxo9ΔF+Neurog2-GFP (*n* = 8) at 6, 12, and 24 hpt. (*F*) Cross-sections of embryos electroporated with the indicated constructs at 24 hpt. The magnified areas are marked with dashed boxes. Closed and open arrowheads indicate persistence and reduced expression of Neurog2-GFP, respectively. (*G*) Well-transfected chicken embryos with the indicated constructs (*n* = 10 per treatment) were subjected to IP with anti-Flag and blotted with anti-Ub or anti-Flag. A total of 20% of the total input was blotted with anti-V5, anti-Flag, and anti-Sox10. Gapdh served as a loading control. (*H*) Densitometric quantification of the levels of Flag-Neurog2 conjugated to Ub in each treatment relative to the control. Hc, heavy chain. Error bars ± SEM (*P < 0.05, **P < 0.01). (Scale bars: embryos, 20 μm; sections, 50 μm.)

**Fig. 8.**
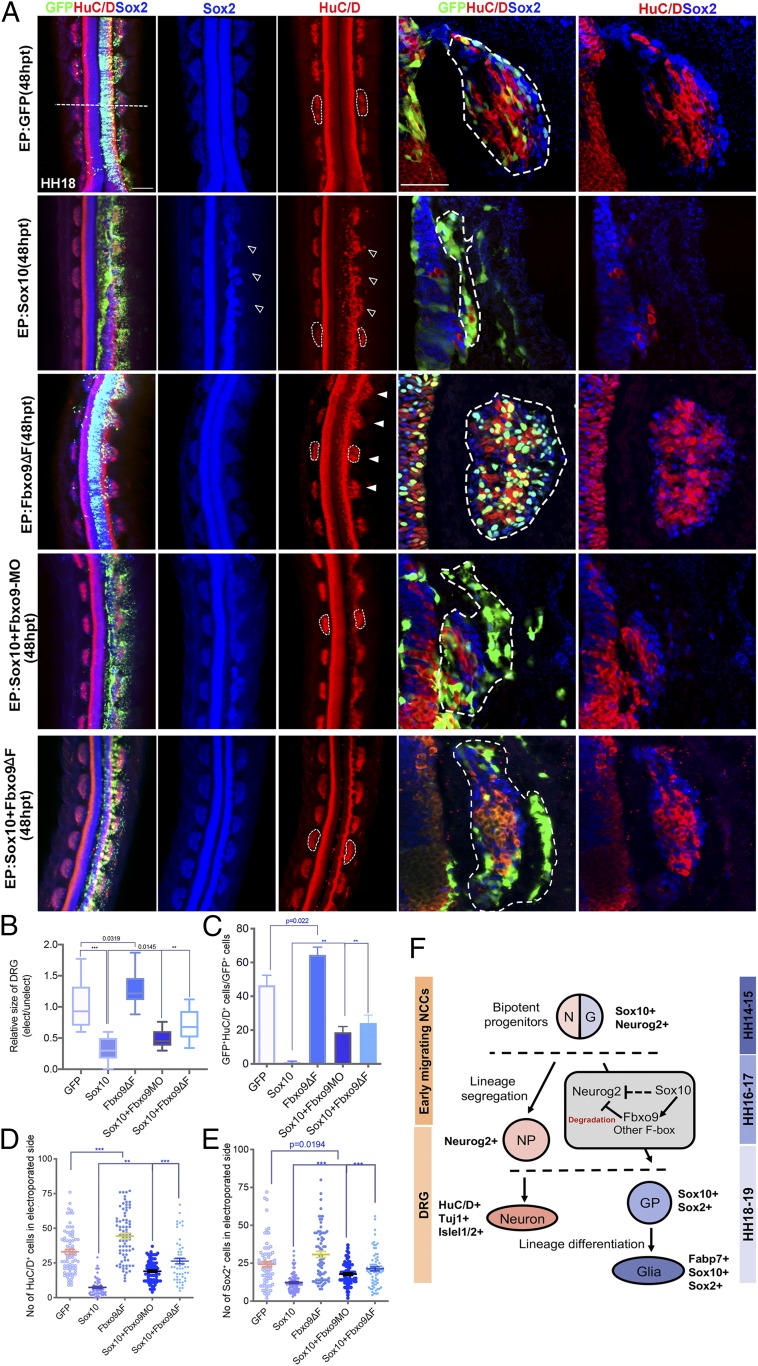
Fbxo9 and possibly other F-box members mediate the role of Sox10 to regulate neuron–glial cell fate choice in the DRG. (*A*) Immunofluorescence for HuC/D and Sox2 of chicken embryos transfected with GFP control (*n* = 7), Sox10 (*n* = 8), and Fbxo9ΔF (*n* = 7). Sox10+Fbxo9ΔF (*n* = 8) and Sox10+Fbxo9 MO (*n* = 8) at 48 hpt. Dotted lines indicate the plane of sectioning and outline the border of the DRG. Open arrowheads indicate DRG dysplasia. Solid white arrowheads indicate enlargement of DRG. (*B*) Graph showing fold differences in the size of the DRG between electroporated and unelectroporated sides of embryos treated with the indicated constructs. (*C*) Quantification of the number of GFP^+^HuC/D^+^ cells in embryos transfected with the indicated constructs. Quantification of the number of (*D*) HuC/D^+^ and (*E*) Sox2^+^ cells in the electroporated side of embryos treated with the indicated constructs. (*F*) Model of the role of Fbxo9 in regulating neuronal–glial cell fate choice in the DRG. Early-migrating bipotent NC progenitors transiently coexpressing Sox10 and Neurog2 undergo lineage segregation processes, in which Sox10 destabilizes Neurog2 (dotted line) through induced expression of Fbxo9 and other F-box factors, resulting in the acquisition of glial progenitor (GP) fate and subsequent differentiation into satellite glial cells, whereas Neurog2^+^ neuronal progenitors (NP) evade Sox10-mediated degradation to differentiate into sensory neurons within the core of the DRG. Error bars ± SEM (**P < 0.01, ***P < 0.001). (Scale bars: embryos, 10 μm; sections, 50 μm.)

As the F-box motif in F-box protein family is responsible for directing the ubiquitination of numerous substrates essential for many cellular functions (31), we examined whether the F-box motif on Fbxo9 was required for regulating Neurog2 protein levels. We generated a mutant form of Fbxo9 lacking the F-box motif (Fbxo9ΔF; Fig. 7*A*), which is able to bind to its substrates without inducing ubiquitination (22). As expected, expression of Neurog2 protein persisted in Sox10^+^ cells overexpressing Fbxo9ΔF in the DRG, whereas Neurog2 was barely detectable in the vector control (Fig. 7*B*). As ectopic Fbxo9ΔF expression was also detected in migratory NCCs where endogenous Fbxo9 was expressed, this raises the possibility that the mutant form of Fbxo9 might act in a dominant-negative manner to antagonize the function of wild-type Fbxo9 through Neurog2 sequestration preventing its degradation. Indeed, coimmunoprecipitation showed that the levels of both endogenous Neurog2 and ectopic Flag-Neurog2 associated with Fbxo9ΔF were higher than with WT Fbxo9 (Fig. 7 *C* and *D*). Consistently, epistasis analysis showed that Neurog2-GFP expression was strongly maintained in embryos transfected with Sox10+Fbxo9ΔF+Neurog2-GFP from 6 to 24 hpt, whereas Neurog2-GFP intensity was robustly diminished in embryos treated with Sox10+Neurog2-GFP (Fig. 7 *E* and *F*). Moreover, the amount of Ub-conjugated Flag-Neurog2 in embryos expressing Flag-Neurog2 alone or SOX10+Flag-Neurog2+Fbxo9ΔF at 9 hpt was barely detectable compared to embryos electroporated with Sox10+Flag-Neurog2, which induced robust ubiquitination of Flag-Neurog2 (Fig. 7 *G* and *H*). These results suggest that overexpression of Fbxo9ΔF could inhibit the ability of Sox10-induced Fbxo9 to degrade Flag-Neurog2 protein. Further examination of embryos electroporated with Fbxo9ΔF at 48 hpt revealed larger sized DRG compared to the untransfected side and the vector control (Fig. 8 *A* and *B*). Consistently, we observed a significant increase in the number of HuC/D^+^ sensory neurons and Sox2^+^ cells, as well as cells coexpressing HuC/D and Sox2 in the DRG of embryos transfected with Fbxo9ΔF compared to the vector control (Fig. 8 *A*, *C*, *D*, and *E*), suggesting expansion of the progenitor pool together with early-onset neurogenesis. Although Sox10-transfected embryos exhibited hypoplastic DRG with a lack of sensory neuron formation, coexpression of Sox10+Fbxo9ΔF restored the size of the DRG to a greater extent than with Sox10+Fbxo9 MO (Fig. 8 *A* and *B*). This was in good agreement with the increase in the number of HuC/D^+^ and Sox2^+^ cells in embryos treated with Sox10+Fbxo9ΔF compared to that of Sox10+Fbxo9 MO (Fig. 8 *A*, *C*, *D*, and *E*). It is possible that Fbxo9ΔF may display broad inhibitory activity on other F-box members induced by Sox10 (Fig. 3*C*). Altogether, these findings suggest that Fbxo9 and probably other F-box members are required to regulate Neurog2 protein stability and to mediate Sox10 in fate determination of neuro-glial progenitors.

## Discussion

Numerous studies have established that Sox10 is functionally important in fate determination of multipotent NC progenitors toward glial versus neuronal cells in the DRG (9–12), but the underlying mechanism of how these two lineages segregate during commitment remains incomplete. In this study, we used RNA-seq to identify Fbxo9 as one of the downstream targets of Sox10 in regulating DRG lineage decisions. Using gain- and loss-of-function approaches in developing chicken embryos, we demonstrated that Fbxo9, an E3 ubiquitin ligase, partly mediated the function of Sox10 to destabilize the sensory determinant Neurog2 in specifying NC progenitors toward glial lineage instead of sensory neurons in the DRG (Fig. 8*F*).

We detected an initial coexpression of Neurog2 and Sox10 in early migrating NCCs before segregation into neuron and glial lineages. The transient expression of proneurogenic Neurog2 could bestow Sox10^+^ NC stem cells with both glial and neurogenic potential as shown previously (16). Indeed, a recent report using single-cell RNA sequencing data combined with spatial transcriptomics and lineage tracing in mouse NCCs revealed that early migrating NCCs expressing Neurog2 before any bifurcation points could form not only sensory neurons but also satellite glial cells in the DRG and in other trunk NC derivatives (32). Thus, early expression of Neurog2 marks NCCs after delamination with broader developmental potential than previously assumed for sensory progenitors (16). In mouse, transcriptional activation of Neurog1/2 depends on Wnt signaling (33), whereas, in zebrafish, Sox10 specifies sensory neuron lineage through regulating neurog1 expression (11). Our data in chick showed that Sox10 overexpression did not affect *Neurog2* mRNA expression, indicating that the regulation of neurogenin expression is species-specific. We found that Sox10 induced the expression of Fbxo9, which is both required and sufficient for regulating Neurog2 protein stability via ubiquitination that was associated with relocation of residual protein from the nucleus to the cytoplasm of Sox10^+^ NCCs. This finding is consistent with previous studies that showed that Neurog2 is a labile protein subject to ubiquitin-mediated proteolysis (17, 19), which contributes to the turnover of many short-lived bHLH proteins both in the cytoplasm and in the nucleus, as shown by MyoD and Ascl1 (26, 34). Thus, our results provide an explanation for the transient expression of Neurog2 protein in Sox10^+^ migratory NCCs.

In agreement with the previous findings of a higher *Sox10* gene dosage inhibiting overt neuronal differentiation of mouse NC stem cells in vitro (4), we also showed that forced expression of Sox10 prevented early migratory avian NCCs from differentiating into neurons and glial cells, leading to a smaller DRG. In addition, the majority of Sox10-expressing cells migrated to the lateral region of the neural tube instead of at the DRG and likely remained undifferentiated, which is consistent with previous observations (5). In contrast, cells overexpressing Fbxo9 migrated to the periphery of the DRG and differentiated into satellite glia, but not sensory neurons, whereas Fbxo9 MO embryos showed the opposite effects. These results suggest that Fbxo9 functions as a regulator of neuron–glial fate choice. As Sox10 regulates Fbxo9 expression, the lack of sensory differentiation in both overexpression studies could partly be attributed to the loss of Neurog2 protein expression. The reason for Sox10 overexpression causing a greater degree of reduction in both neuron and glial lineages than for Fbxo9 could be due to multiple transcriptional targets of Sox10 that regulate not only glial cell specification (9) but also the maintenance of multipotent NC stem cells and NC migratory behavior (4, 27). Thus, elevated expression of Sox10 might affect multiple aspects of NC development. In addition to Neurog2, it is possible that Fbxo9 regulates glial differentiation and specification of other NC derivatives through its association with different substrates, which remain to be identified and characterized.

The finding that the F-box motif in Fbxo9 is required to effectively reduce Neurog2 protein levels by ubiquitination prompted us to suggest that Fbxo9ΔF functions in a dominant-negative manner through Neurog2 sequestration preventing degradation by both endogenous and ectopic Fbxo9 expression. As our RNA-seq analysis identified other members of the F-box family and some of them were expressed at low levels in NCCs, it is possible that Fbxo9ΔF also exerts dominant-negative effects on their functions. In agreement with this, forced expression of Fbxo9ΔF alone and together with Sox10 resulted in greater increases and restoration of sensory neuron formation, respectively, when compared to the Fbxo9 MO group. In addition, we also observed more cells coexpressing Sox2 and HuC/D in the DRG of embryos treated with Fbxo9ΔF than in those treated with Fbxo9 MO. These findings suggest that, besides Fbxo9, other F-box proteins might also be involved in neuron–glial lineage decision. It has been shown that Sox2 marks bipotent neuron–glial progenitors and satellite glial cells but not sensory neurons in the DRG (25, 35). However, ablation of Sox2 function in NCCs revealed that it is an essential requirement for sensory neurogenesis (36). Whether glial fate is affected in vivo was not examined in this study. These data underlie the complexity of the function of Sox2 in determining neuronal lineage and possibly glial differentiation. Thus, the detection of cells coexpressing Sox2 and HuC/D in both Fbxo9MO and Fbxo9ΔF embryos suggests early acquisition of neuronal fate in the expanded pool of bipotent progenitors. Moreover, this is likely due to persistence of Neurog2 protein in Sox10^+^ NCCs, which further indicates the essential role of Fbxo9 and possibly other F-box members in orchestrating proper lineage segregation between sensory neurons and satellite glial cells during commitment. Further studies are needed to elucidate whether other F-box members are involved in this process.

Although several studies have documented the importance of ubiquitin-mediated protein degradation in regulating NCC fate determination and melanocyte formation (37–39), whether this process also plays a role in DRG lineage decisions is not known. Our studies demonstrated that degradation of neuronal determinant Neurog2 by Sox10-induced Fbxo9-mediated proteolysis is a major contributor in directing multipotent NC progenitors toward glial lineage instead of sensory neurons in the forming DRG. Recent studies revealed that satellite glial cells play active roles in chronic pain (40). Therefore, understanding how protein degradation contributes to the specification of satellite glial cells will lead to a better understanding of the etiology of such disorders.

## Materials and Methods

Fertilized chick eggs obtained from Jinan Poultry (Tin Hang Technology) were incubated at 38 °C in a humidified incubator. Embryos were staged according to Hamburger and Hamilton (HH) stages (41). All animal experiments were approved by the committee on the use of live animals in teaching and research of The University of Hong Kong. In ovo electroporation was carried out as described previously (27). Morpholinos were purchased from Gene Tools (https://www.gene-tools.com/). Detailed protocols regarding the generation of various constructs, the generation of Sox10NGFP mutant mice, RNA sequencing, qPCR, in situ hybridization, immunofluorescence, immunoprecipitation, Western blot, and statistical analysis are provided in *SI Appendix*, *SI Materials and Methods*. The RNA sequencing data and list of up- and down-regulated genes by Sox10 overexpression have been deposited in Figshare (11378727) (29).

## Supplementary Material

Supplementary File

Supplementary File

Supplementary File
