# Supplementary material for: Fbxo9 functions downstream of Sox10 to determine neuron-glial fate choice in the dorsal root ganglia through Neurog2 destabilization
Source: Proc Natl Acad Sci U S A. 2020 Feb 6;117(8):4199–210. doi: 10.1073/pnas.1916164117 (PMC7049171; doi:10.1073/pnas.1916164117)
Supplement: Supplementary File [file pnas.1916164117.sapp.pdf]

Fbxo9 functions downstream of Sox10 to determine neuron-glial fate choice in the dorsal root ganglia through Neurog2 destabilization

Jessica Aijia Liu, Andrew Tai, Jialin Hong, May Pui Lai Cheung, Mai Har Sham, Kathryn S.E. Cheah, Chi Wai Cheung and Martin Cheung

Corresponding author: Dr. Martin Cheung
Email: mcheung9@hku.hk

www.pnas.org/cgi/doi/10.1073/pnas.1916164117

SI Materials and Methods

Expression vectors and morpholinos.

Full length chick Sox10, Sox9, and Fbox9 cDNA, and Fbxo9ΔF were inserted upstream of an internal ribosomal entry site (IRES) and a nuclear localization sequence (nls)-tagged EGFP in a pCIG (a gift from A. McMahon, University of Southern California, USA) or tdTomato vector. Neurog2 in GFP-N1 was a gift from P. Scotting, The University of Nottingham, United Kingdom; and Flag-Neurog2 in pCIG-IRES-nls-EGFP was a gift from S. Bel-Vialar, Université de Toulouse, France.

Morpholinos (MO) for Sox10, Fbxo9 and corresponding MO-controls (Ctrl) tagged with 3′ fluorescein or 3'-Lissamine were obtained from Gene Tools with the following sequences:

Sox10-MO	5′-CCGACAGATCTTGGTCATCAGCCAT-3′
Sox10-MO-Ctrl	5′-CCCACACATCTTGCTCATGACCCAT-3′
Fbxo9-MO	5′-AATCTTCTTCAGCTTCTGCCATGCT-3′
Fbxo9-MO-Ctrl	5′-AGAATCTTGTTCACCTTCTCCGATG-3′

The final molar concentration of each morpholino oligonucleotide was 0.75 mM mixed in V5-EGFP as the carrier DNA for in ovo electroporation.

Chick embryos and electroporation.

Fertilized chick eggs obtained from Jinan Poultry Co. (Tin Hang Technology) were incubated at 38°C in a humidified incubator. Embryos were staged according to Hamburger and Hamilton (HH) stages (1). All animal experiments were approved by the Committee on the Use of Live Animals in Teaching and Research, The University of Hong Kong (CULATR No: 3550-15). In ovo electroporation was performed as described previously (2). Plasmid DNA or morpholinos were

injected into the lumen of neural tubes at HH11–12, electrodes were placed on either side of the neural tube, and electroporation was carried out using a BTX electroporator delivered as five 50-ms pulses at 33 V. Electroporated embryos were allowed to develop for 24 or 48 h post-transfection (hpt) before being processed for Western blotting, immunofluorescence, in situ hybridization, and immunoprecipitation.

Mice

To generate *Sox10NGFP* mutant mice, the N terminal domain of Sox10 was fused to an EGFP reporter (3). Targeted ES cell clones were injected into blastocysts of C57BL/6J obtained from the Laboratory Animal Unit of The University of Hong Kong. Mutant mice were backcrossed with C57BL/6J to maintain the colony. All animal experiments were performed in accordance with procedures approved by the Committee on the Use of Live Animals in Teaching & Research, The University of Hong Kong (CULATR No. 2131-10 and 3120-13).

FACS and RNA sequencing

After electroporation, embryos were incubated at 37°C until HH13. Embryos with weak GFP expression or at incorrect stages of development were discarded, and only embryos that showed robust GFP expression were harvested. The trunk showing strong GFP expression in the neural tube was dissected in cold PBS under fluorescent stereoscope. Tissue samples were pooled together and cells were dissociated with Dispase/Trypsin. Cells were washed and passed through a 40-μm cell strainer (BD), and resuspended in FACS buffer at 1×10^5 cells/mL. The GFP^+ cells were enriched using BD FACSAria I Cell Sorter (BD Biosciences) with 7-AAD exclusion to eliminate dead/damaged cells. Approximately 50,000 GFP^+ cells per replicate were collected for

RNA extraction using RNAqueous-Micro Kit (Ambion) and genomic DNA was removed by TURBO DNase treatment for 30 min. RNA quality was assayed in an Agilent 2100 Bioanalyzer and samples with an RNA integrity number (RIN) >8 were further processed. High-quality RNA (~50 ng) was used for RNA amplification and cDNA synthesis. The cDNA with adapter was further amplified to make a library of single stranded circular DNA, which were used to generate DNA nanoballs (DNBs) by rolling circle replication for the enhancement of fluorescence signals during the sequencing process. The DNBs were loaded into the patterned nanoarrays for 100-bp pair-end sequencing in a BGISEQ-500 platform. Biological replicates were used for each condition (GFP vector control and Sox10 overexpression). Hierarchical clustering analysis was performed using cluster software and Euclidean distance matrix as a measure of similarity.

RT-quantitative real-time PCR

Total RNA of the sorted cells was extracted using MiniBEST Universal RNA extraction Kit (Takara) and reverse transcribed for cDNA synthesis using PrimeScript RT Master Mix (Takara). qPCR reactions were carried out in triplicate on StepOne Plus Real-time PCR system (Applied Biosystem) using SYBR Premix Ex Taq II (Takara). Chick Gapdh was used as control. All experimental values were normalized to those obtained for Gapdh. The data are expressed as fold change relative to the control GFP which is set to 1 and represent mean standardised values ± S.E.M. List of primer sequences for the genes under evaluation are shown in Table S1.

In situ hybridization and immunofluorescence

Electroporated embryos were harvested and fixed for 1 h at 4°C in 4% paraformaldehyde (PFA) in 0.1 M phosphate buffer (PB). For immunohistochemistry, embryos were cryoprotected with

30% sucrose in PB and sectioned at 10-μm thickness, and localization of proteins was performed as described previously (4). For whole-mount immunofluorescence, embryos were fixed for 2 h in 4% PFA and then twice blocked with 5% fetal bovine serum in PBS in 1% Triton for 1 h. Embryos were incubated with primary antibodies for 2 days, followed by incubation overnight with secondary antibodies. The antibodies included sheep anti-GFP (1:1000; AbD Serotec 4745-1051), rabbit anti-GFP (1:1000; Rockland 600-401-215), guinea pig anti-Neurog2 (1:32000, gift from B. Novitch), goat anti-Sox2 (1:500; R&D AF2018), goat anti-Sox10 (1:500; R&D AF2864), mouse anti-HuC/D (1:1000; Thermo Fisher Scientific A-21271), mouse anti-V5 (1:1000, Thermo Fisher Scientific R960-25) and mouse anti-Islet1/2 (1:100, DSHB 39.4D5). A Carl Zeiss LSM 710 and 800 Meta laser scanning confocal microscopes were used to acquire images. In situ hybridization was performed using NBT/BCIP detection kit (Roche). The following anti-sense chick RNA probes were used: *Fbxo9*, *Fbxo2*, *Fbxo25*, *Fbxw11*, *Fabp7* and *Sox10 3'UTR (5).*

Immunoprecipitation and Western analysis

Ten well-transfected chicken neural tubes were micro-dissected and lysed using Pierce MS-compatible magnetic IP kit (90409). Pre-clearing of embryonic neural tube lysates was performed by incubating with protein A/G magnetic beads for 16 h at 4°C, followed by overnight immunoprecipitation with 4 μg mouse anti-Flag-M2 (Sigma), mouse V5 (Invitrogen) or mouse anti-IgG as the negative control. The protein-antibody complex was incubated with protein A/G magnetic beads for 4 h at 4°C. The immunoprecipitated protein complex was eluted using 4×SDS sample loading buffer. For the Western blot analysis, immunoprecipitated protein complex and total input of protein lysate or total protein lysate from different electroporated constructs were immunoblotted with mouse anti-Flag-M2 (1:3000), rabbit anti-Neurog2 (Bioss bs-3692R), goat

anti-Sox10 (1:500), mouse anti-V5 (1: 1500, Invitrogen), mouse anti-ubiquitin (1:1000, Millipore 05-944), rabbit anti-Fbxo9 (1:1000), and rabbit anti-Gapdh (1:3000; Santa Cruz). Quantification of protein bands intensity on Western blots were conducted by Image J.

Statistical analysis

Data from at least three separate experiments were presented as means ± SEM. *P* values were calculated using Student's *t* test and one-way analysis of variance. A value of $P < 0.05$ was considered statistically significant.

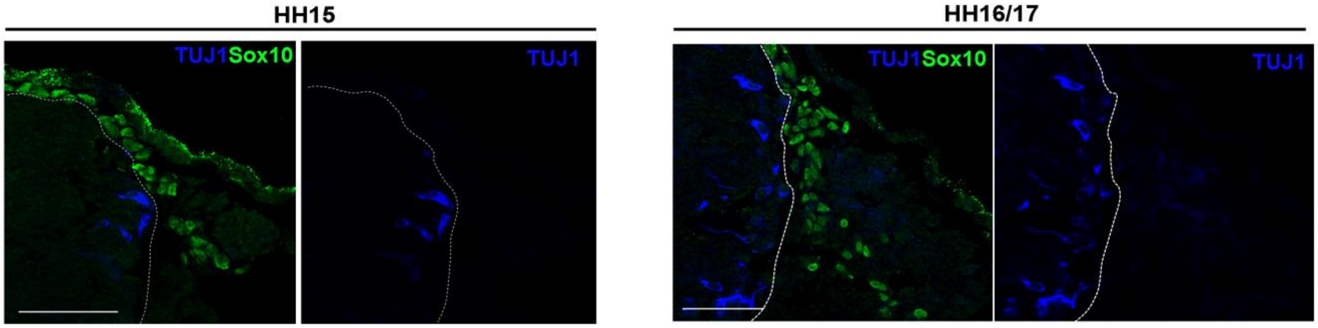
HH15
TUJ1Sox10
TUJ1
HH16/17
TUJ1Sox10
TUJ1

Fig. S1. Lack of TuJ1 expression in migratory NCCs from HH15-17 chick embryos. Immunofluorescence for Sox10 and TuJ1 in the transverse sections of the trunk neural tube from chick embryos at HH15 and HH16/17. Scale bar: 50μm.

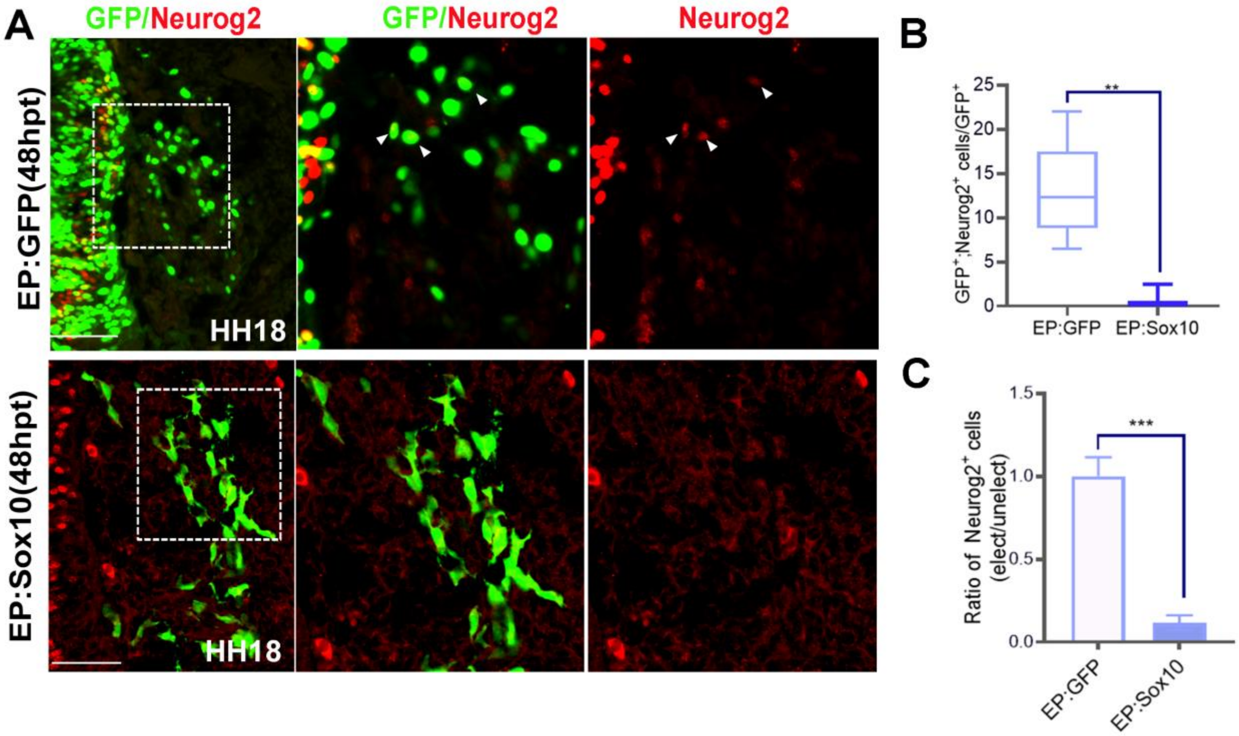
A
GFP/Neurog2
GFP/Neurog2
Neurog2
EP:GFP(48hpt)
HH18
EP:Sox10(48hpt)
HH18
B
GFP+;Neurog2+ cells/GFP+
**
25
20
15
10
5
0
EP:GFP
EP:Sox10
C
Ratio of Neurog2+ cells (elect/unelect)
***
1.5
1.0
0.5
0.0
EP:GFP
EP:Sox10

Fig. S2. Overexpression of Sox10 downregulates Neurog2 protein expression at 48 hpt. Immunofluorescence for Neurog2 on transverse sections of embryos electroporated with the indicated constructs at 48 hpt. The magnified areas are marked with dotted squares. White solid arrowheads indicate detection of endogenous Neurog2 expression in cells expressing vector control. (B) Quantification of the number of $GFP^{+}Neurog2^{+}$ in the total number of GFP^{+} cells in embryos treated with the indicated constructs. (C) Graph showing ratio of $Neurog2^{+}$ cells between the electroporated and unelectroporated sides of embryos treated with the indicated constructs. Error bars ± S.E.M. **p<0.01, ***p<0.001. Scale bar: 50 μm.

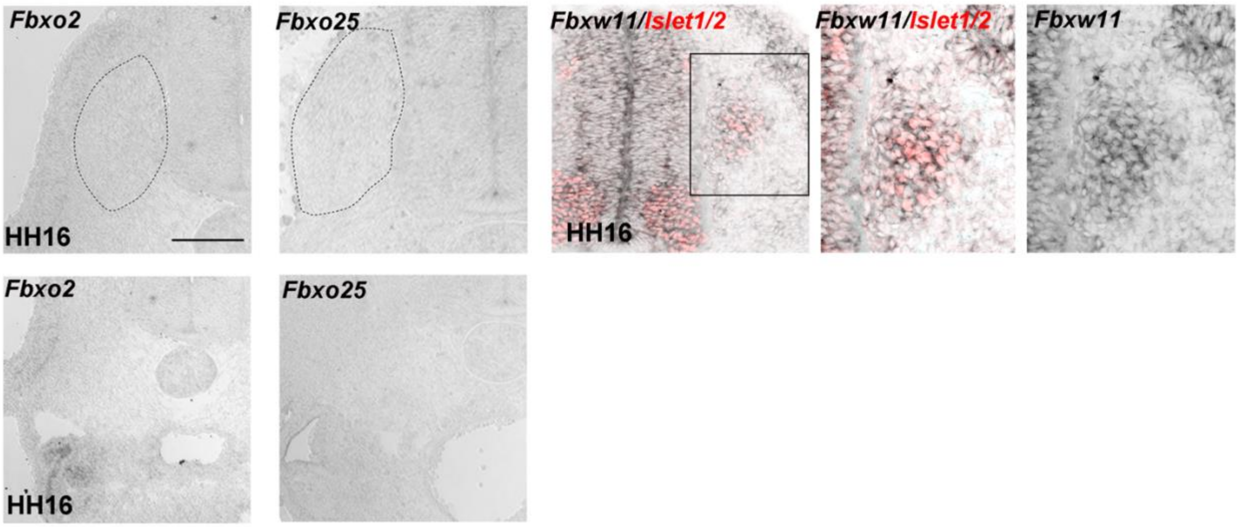
Fbxo2
HH16
Fbxo25
Fbxw11/Islet1/2
HH16
Fbxw11/Islet1/2
Fbxw11
Fbxo2
HH16
Fbxo25

Fig. S3. Expression of other F-box family members in the DRG. In situ hybridization for *Fbxo2*, *Fbxo25* and *Fbxw11* on transverse sections of HH16 chick embryos. Immunofluorescence for Islet1/2 was conducted on sections stained with *Fbxw11*. The magnified area is marked with black box. Dotted lines outline the border of the DRG. Scale bar: 50 μm.

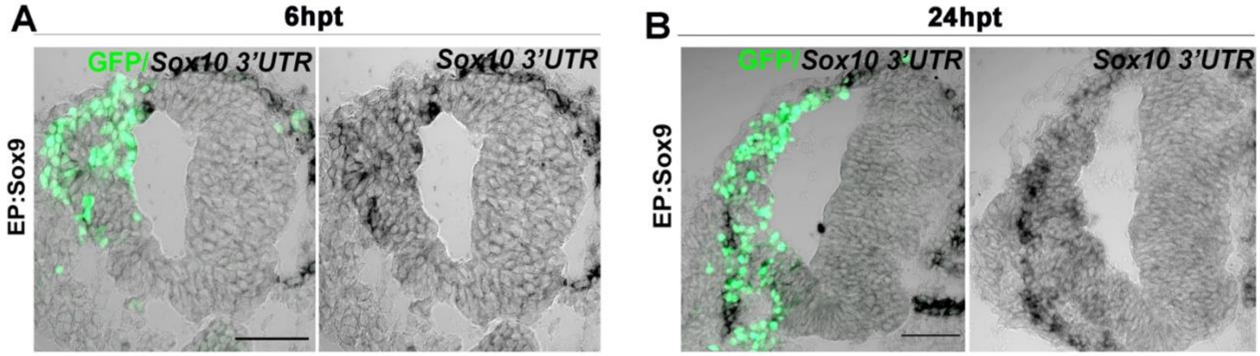
A
6hpt
GFP/Sox10 3'UTR
Sox10 3'UTR
EP:Sox9
B
24hpt
GFP/Sox10 3'UTR
Sox10 3'UTR
EP:Sox9

Fig. S4. Misexpressed Sox9 induces weak ectopic Sox10 expression in the developing chick neural tube at 6 and 24 hpt. In situ hybridization for anti-sense probe targeting *Sox10 3'UTR (3' untranslated region)* to avoid detection of ectopic *Sox9* expression on cross-sections of embryos electroporated with Sox9 at 6 (n=5) (A) and 24 hpt (n=6) (B). Scale bar: 50 μm.

Table S1. List of qPCR primer sequences

Genes	Forward primer sequences	Reverse primer sequences
Sox10	GAGGAAATTGGCTGACCAGTA	CCGCTCTGCCTCTTCAATAA
Dlx5	GAACGGCAAGCCAAAGAAAG	GAGGTATTGGGTCTTCTGGAAC
Dlx3	CTATTCCTACAGCCCGTCCTA	TGCGGATCTTCTTGGGTTTC
Tfap2a	GAGGAGGGCAAAGTCCAAA	AGGTGAGCAAGGTGACATTAG
Hand1	AACAAGAGGAAACGGGAGAC	TCAGTTCCAGAGCCCAGA
Ednrb	GGTGGACTGCTGTTGAAATTG	CAGGCAGATTCTGAGATCCTTT
Fbxo9	CTTGTCCCTGACATCGAGTTTA	CTGACAGGAGATCAGCCATTT
Fbxo2	GATCAGAATCCTGGCTTCCATAC	CCAAAGAGCAGCTCCATCAA
Fbxo25	GTATGGAGACACCTTGCATTTC	CTGAGGAGATACTGGCATGAG
Fbxw11	GGCATCACCTACTGACATCAC	ACCTGATGCCGACACAATATAC
Shh	TTCTCTTGGTGGGCTTCATC	CTCTGCCACATTGGGAATAAAC
Hhip	CCCTCGACGATATGGAAGAAAT	TGCTCCGTGGTATGGAATAAG
Gabra4	TGATGGTGGGAGTCGAATTG	GTGAAAGGGTTGGGACTTGA
Gabrg2	CAACTGAGGTGGTGAAGACTAC	GCAGGGAATGTAGGTCTGAATAG
Gad67	ACCTGTATGCCCGAGATTTG	AACGTCTTCCTCACGTAGTTG

Dataset S1: RNA-seq dataset normalized to the control

Dataset S2: List of up- and downregulated genes by Sox10 overexpression

References

1. V. Hamburger, H. L. Hamilton, A series of normal stages in the development of the chick embryo. 1951. *Dev Dyn* **195**, 231-272 (1992).
2. J. A. Liu *et al.*, Asymmetric localization of DLC1 defines avian trunk neural crest polarity for directional delamination and migration. *Nat Commun* **8**, 1185 (2017).
3. J. A. Liu *et al.*, Identification of GLI Mutations in Patients With Hirschsprung Disease That Disrupt Enteric Nervous System Development in Mice. *Gastroenterology* **149**, 1837-1848 e1835 (2015).
4. M. Cheung *et al.*, The transcriptional control of trunk neural crest induction, survival, and delamination. *Dev Cell* **8**, 179-192 (2005).
5. J. A. Liu *et al.*, Phosphorylation of Sox9 is required for neural crest delamination and is regulated downstream of BMP and canonical Wnt signaling. *Proc Natl Acad Sci U S A* **110**, 2882-2887 (2013).
